# Acquisition of bedaquiline and clofazimine resistance in association with a novel loss-of-function mutation in the *pepQ* gene during treatment of multidrug-resistant tuberculosis

**DOI:** 10.1128/asmcr.00126-25

**Published:** 2025-11-12

**Authors:** Melissa Richard-Greenblatt, Ruchika Bagga, Carla Duncan, Maxime J. Billick, Howard Song, Natasha F. Sabur, Vincent Escuyer, Karen Lam, Sarah K. Brode

**Affiliations:** 1 Public Health Ontario 153300, Toronto, Ontario, Canada; 2 Hospital for Sick Children, Toronto, Ontario, Canada; 3 Department of Laboratory Medicine and Pathobiology, University of Toronto 7938, Ontario, Canada; 4 Department of Infectious Diseases, University of Toronto 7938, Ontario, Canada; 5 University Health Network 7989, Toronto, Ontario, Canada; 6 Division of Respirology, University of Toronto 7938, Toronto, Ontario, Canada; 7 Unity Health—St. Michael’s Hospital, Toronto, Ontario, Canada; 8 Wadsworth Centre, New York State Department of Health 1094, Albany, New York, USA; Rush University Medical Center, Chicago, Illinois, USA

**Keywords:** Canada, clofazimine resistance, bedaquiline resistance, *pepQ*, tuberculosis, XDR-TB, MDR/RR-TB

## Abstract

**Background:**

Bedaquiline (BDQ) has transformed the management of multidrug-resistant (MDR) and rifampin-resistant tuberculosis (TB). Unfortunately, the expanded use of BDQ in these regimens has been accompanied by resistance, which is steadily increasing in certain regions of the world. Nonetheless, our understanding of the mechanisms behind BDQ resistance remains poor, limiting the utility of more rapid molecular or genomic-based diagnostics for the detection of BDQ-resistant isolates.

**Case Summary:**

We describe an unusual case of a rapid, 2-year evolution of a fully susceptible *Mycobacterium tuberculosis* strain to extensively drug-resistant TB in a 44-year-old Canadian-born woman with Crohn’s disease. Comparative whole-genome sequencing captured the progressive development of resistance mutations and identified a novel loss-of-function mutation (Glu-177-STOP) in the *M. tuberculosis pepQ* gene that was associated with treatment failure while on BDQ and phenotypic BDQ/clofazimine (CFZ) cross-resistance. Therapeutic drug monitoring while on MDR therapy (daily ethambutol, pyrazinamide, linezolid, CFZ, and intravenous amikacin) detected low serum levels of CFZ, which was not addressed prior to the addition of BDQ to her 5-drug regimen and may have selected for BDQ/CFZ cross-resistance.

**Conclusion:**

This case contributes to the limited clinical data implicating *pepQ* in BDQ/CFZ cross-resistance and describes a novel loss-of-function mutation associated with resistance. As our understanding of genotypic BDQ resistance remains elementary, when novel drug mutations arise, practitioners should consider their significance in the context of phenotypic drug susceptibility test results and the patient’s clinical response.

## INTRODUCTION

The emergence of multidrug-resistant tuberculosis (MDR-TB) has impaired global tuberculosis (TB) control efforts. Historically, MDR-TB therapy was limited by the lack of new effective medications and warranted the use of at least five drugs, including injectable agents, for a total of at least 18 months (1). These treatments were associated with high rates of unfavorable outcomes due to poor tolerance, mortality, and high rates of failure/recurrence (2).

Bedaquiline (BDQ) terminated a 45-year deadlock in TB drug discovery, revolutionizing MDR/rifampin-resistant (RR)-TB treatment. The World Health Organization (WHO) introduced BDQ into MDR-TB management in 2013 (3) and revised second-line drug classifications for 18-month regimens, improving effectiveness and safety (4). Further trials investigating BDQ-containing regimens led to novel all-oral, 6-month BPaLM/BPaL (BDQ [B], pretomanid [P], linezolid [L] ± moxifloxacin [M]) and 9-month (BDQ, moxifloxacin/levofloxacin, ethionamide, ethambutol, high dose isoniazid, pyrazinamide, and clofazimine [CFZ]) regimens recommended by the WHO for MDR/RR-TB treatment in 2022 (5). These newly recommended regimens can dramatically increase cure rates due to higher efficacy (6–8), allow broader access due to lower cost (9), and improve patient quality of life as they are all-oral and significantly shorter than conventional treatment approaches.

BDQ resistance is one of the biggest threats to the successful treatment of MDR-TB. Mutations of *atpE* (*Rv1305*), *pepQ* (*Rv2535c*), and *mmpR* (*rv0678*) have been associated with phenotypic resistance to BDQ, while mutations of the latter two genes have been implicated in cross-resistance to CFZ and BDQ (10–13). Nonetheless, due to limited drug susceptibility testing (DST) capacity and BDQ’s recent introduction, mechanisms underlying resistance are poorly understood. Here, we report a novel *pepQ* gene mutation associated with BDQ and CFZ cross-resistance in a 44-year-old woman with MDR-TB and Crohn’s disease.

## CASE PRESENTATION

**Fig 1:**
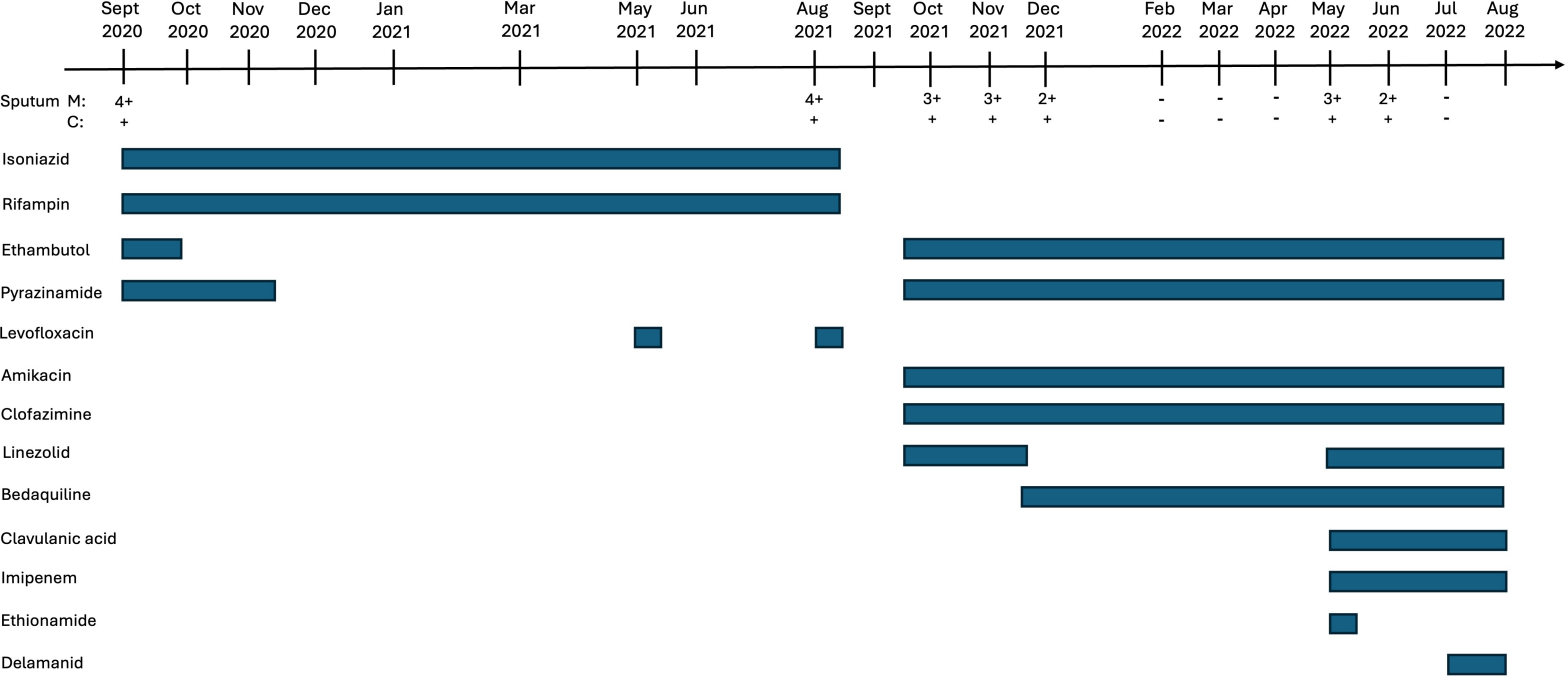
Timeline of treatment course and microbiologic data for a patient with Crohn’s disease developing XDR-TB from a fully susceptible isolate. Solid bars correspond to the treatment period for the respective antibiotics. M, acid-fast bacilli microscopy smear result; C, culture result for *M. tuberculosis*.

A 44-year-old Canadian-born woman was transferred to our TB inpatient service with MDR-TB in August 2021. Initially diagnosed with cavitary TB disease in September 2020, her sputum cultures grew drug-susceptible *Mycobacterium tuberculosis* (Fig. 1), and she was started on standard first-line therapy: isoniazid (300 mg), rifampin (600 mg), pyrazinamide (1,000 mg), and ethambutol (800 mg) as per the Canadian TB Standards (14). Her only known TB exposure was at age 10 when her great-grandmother had pulmonary TB. She denied social risk factors for TB and had a negative single-step tuberculin skin test in 2017. Her medical history was significant for Crohn’s disease (diagnosed in 1998) necessitating prior bowel resections, and chronic diarrhea likely attributed to short bowel syndrome. In February 2020, she was advised by her care team to discontinue adalimumab and methotrexate as they were deemed ineffective, and she was being considered for an alternative biologic. However, due to abdominal pain, poor oral intake, and weight loss, she was started on a tapering prednisone regimen (40 mg daily) in February 2021, which resulted in symptom improvement. She completed 6 months of recommended TB therapy in March 2021.

In May and August 2021, she received week-long courses of levofloxacin (500 mg daily) prescribed by outside providers for presumed bronchitis. Sputum was collected in August 2021 for mycobacteriology work-up and returned 4+ AFB, and cultures grew *M. tuberculosis*. Phenotypic DST demonstrated resistance to rifampin, isoniazid, and moxifloxacin (Table 1).

**TABLE 1:** Evolution of resistance in *M. tuberculosis* isolates cultured from repeated sputum samples in a patient with Crohn disease^*a*^

Specimen collection date	Result for tested drug^*b*^:															
	H	Hh	R	E	Z	Mfx	Am	Cm	Km	S	Eto	Lzd	PAS	Rfb	CFZ^*c*^	BDQ
September 2020	S	S	S	S	S	–^*d*^	–	–	–	–	–	–	–	–	–	–
August 2021	R	R	R	S	S	R	S	S	S	S	S	S	S	R	–	–
November 2021	R	R	R	S	S	R	S	S	S	S	S	S	S	R	WT	S
May 2022	R	R	R	S	S	R	S	S	R	S	S	S	S	R	NWT	R

^*a*^ *M. tuberculosis* susceptibility methods varied depending on the drugs tested. Susceptibility to first-line drugs (H, Hh, R, E, Z) and S was determined using BD SIRE AST, BD INH 0.4 mg/L, and PZA AST kits on the MGIT 960 systems, according to manufacturer’s protocols. Second-line drugs, except BDQ and CFZ, were also incubated on the MGIT 960 system; however, drugs were manually prepared. BDQ and CFZ susceptibility testing was performed using microbroth dilution methods as previously described (15). Susceptibility interpretations are based on CLSI M62 1st edition breakpoints where applicable. Regardless of the breakpoints used (WHO endorsed critical concentration or EUCAST) for BDQ, the interpretations remain the same for BDQ.

^*b*^ H, isoniazid (0.1 mg/mL); Hh, high-dose isoniazid (0.4 mg/mL); R, rifampin; E, ethambutol; Z, pyrazinamide; Mfx, moxifloxacin; Am, amikacin; Cm, capreomycin; Km, kanamycin; S, streptomycin; Eto, ethionamide; Lzd, linezolid; PAS,* p*-aminosalicylic acid; Rfb, rifabutin; CFZ, clofazimine; WT, wild type; NWT, non-wild type; and BDQ, bedaquiline.

^*c*^ There are currently no breakpoints described for CFZ, and our interpretation is based on the epidemiological cutoff value of 0.25 mg/L (15).

^*d*^ – indicates that phenotypic drug susceptibility testing was not performed for the drug on the respective date.

In September 2021, the patient was transferred to our TB service and initiated on daily ethambutol (700 mg), pyrazinamide (1,000 mg), linezolid (600 mg), CFZ (100 mg), and intravenous amikacin (300 mg). Significant nausea and vomiting occurred with the introduction of these antibiotics. Therapeutic drug monitoring (TDM) was performed in November 2021, and all drugs were in the therapeutic range except for CFZ. Given poor gastrointestinal tolerance and overall improvement in her status, no changes were made to the CFZ dose. BDQ (400 mg daily for 2 weeks and then 200 mg 3× weekly) was added to her existing 5-drug regimen once approval from the Health Canada Special Access Program was obtained in November 2021. She experienced ongoing nausea and vomiting likely due to serotonin syndrome from linezolid and citalopram interactions, leading to linezolid discontinuation. In December 2021, improvements were observed on chest X-ray compared to November 2021 imaging. Sputum smear and culture conversion was achieved 4.5 months after initiating the MDR treatment regimen, and her symptoms nearly resolved. Monthly follow-up sputum cultures remained negative, and chest X-rays were stable from February to April 2022.

In May 2022, the patient described an increasingly productive cough and night sweats. She was re-admitted to the TB unit to augment treatment with intravenous imipenem, oral clavulanic acid, and linezolid. A sputum sample was sent and returned as 3+ AFB. Repeat phenotypic DST on the new *M. tuberculosis* isolate revealed resistance to isoniazid, rifamycins, moxifloxacin, and kanamycin (Table 1). Lack of access to phenotypic DST for BDQ and CFZ in Canada at this time led us to perform whole-genome sequencing (WGS) to identify genotypic markers associated with resistance. In the May 2022 isolate, we detected a novel *pepQ* mutation (Glu-177-STOP) with unknown significance. WGS of the patient’s prior isolates did not identify any *pepQ* or other known mutations associated with BDQ and CFZ resistance. To determine if the patient’s TB treatment failure was due, in part, to BDQ and CFZ resistance, we forwarded the *M. tuberculosis* isolates from November 2021 and May 2022 to the Mycobacteriology Laboratory at the Wadsworth Center, New York State Department of Health, for phenotypic DST and confirmatory genotypic resistance testing.

Delamanid was added to the regimen in July 2022 immediately following procurement, but unfortunately, the patient did not improve. She passed away from acute respiratory distress syndrome and progressive pulmonary disease in August 2022. Shortly thereafter, the Wadsworth Centre confirmed that the *pepQ* (Glu-177-STOP) mutation was only detected in the most recent isolate, as was phenotypic resistance to BDQ and CFZ based on microbroth dilution methods (Table 1).

## DISCUSSION

Through phenotypic DST and *M. tuberculosis* WGS, we monitored the evolution of resistance from a pan-susceptible TB isolate to an XDR-TB strain over a 2-year period, and we identified a novel loss-of-function mutation in the *pepQ* gene associated with phenotypic BDQ/CFZ cross-resistance and treatment failure. Clinical *M. tuberculosis* isolates with *pepQ* mutations associated with phenotypic BDQ resistance are rarely described (16), and frequently, those observed are not linked to BDQ and CFZ resistance (17–19). Loss-of-function mutations in *pepQ* were added to the 2nd edition of the WHO catalog of *M. tuberculosis* complex mutations in 2023 but are given an interim association with resistance due to limited data (20). Prior to our case, only a single study in a murine model had previously reported that *pepQ* loss-of-function mutations confer low-level resistance to BDQ and CFZ (10). While the mechanism of resistance to both drugs remains unclear, *in vitro* studies demonstrating the reversion of BDQ resistance of a *pepQ* mutant to wild type in the presence of efflux pump inhibitors suggest drug efflux as a mechanism of resistance (10).

Mutations in *mmpR* and *atpE* are also associated with phenotypic resistance to BDQ (10–13), with ~80% of acquired resistance mutations occurring in the *mmpR* gene (21–24). MmpR is a transcriptional repressor of the efflux pump system, MmpS5-MmpSL5. Mutations leading to its partial or complete inactivation result in low-level resistance to BDQ and CFZ due to enhanced efflux of these drugs (12). In contrast, mutations in BDQ’s target, *atpE*, which encodes for the c-subunit of the ATP synthase, are rarely described in clinical strains likely due to higher fitness costs (16, 25). Interestingly, both *mmpR* and *pepQ* mutants were found to be preferentially selected for and able to grow in mice treated with BDQ over *atpE* mutants, suggesting that these mutations offer a better balance between fitness maintenance and reduced BDQ susceptibility (10), yet *pepQ*-mediated BDQ resistance in clinical isolates is rare (16).

As seen in our patient, resistance to BDQ can be acquired while on therapy (26, 27). A systematic review reported a 2.2% median proportion of patients on BDQ-containing regimens developing phenotypic resistance (26). However, resistance to BDQ has also been reported in BDQ treatment-naive patients with MDR-TB disease (16, 28). Primary resistance rates vary considerably by country (28), with the highest rates described in South Africa (8%) (16). These findings emphasize the importance of phenotypic DST for BDQ resistance when starting a BDQ-containing regimen as well as monitoring for emerging resistance in patients with delays in culture conversion.

The rapid development of resistance to a multitude of drugs in our patient was likely driven by several factors. She had extensive cavitary pulmonary TB, which may have led to lower drug concentrations within cavities (29). Additionally, low serum drug concentrations due to pharmacokinetic variability (30), drug malabsorption associated with her Crohn’s disease, and/or significant intermittent vomiting may have also impacted drug levels in this patient. TDM was only performed while on MDR-TB therapy, and initially detected low levels of CFZ, which were not addressed. It is plausible that low levels of CFZ may have selected for its resistance and, as a by-product, BDQ resistance as well. Intermittent use of levofloxacin for the empiric treatment of bronchitis may have also led to fluoroquinolone resistance in this patient.

In conclusion, our findings add to the limited clinical data implicating *pep*Q in BDQ/CFZ cross-resistance and describe a novel loss-of-function mutation associated with resistance. As our understanding of genotypic BDQ resistance remains elementary, when novel drug mutations arise, practitioners should consider their significance in the context of phenotypic DST results and the patient’s clinical response.

## Data Availability

Whole-genome sequences of the four *Mycobacterium tuberculosis* isolates described in this case are available in the European Nucleotide Archive (ENA) under project PRJEB101432. Individual isolate accession numbers are as follows: September 2020 (SAMEA120422717), August 2021 (SAMEA120422718), November 2021 (SAMEA120422719), and May 2022 (SAMEA120422720).
